# Supplementary material for: Reconciling Mining with the Conservation of Cave Biodiversity: A Quantitative Baseline to Help Establish Conservation Priorities
Source: PLoS One. 2016 Dec 20;11(12):e0168348. doi: 10.1371/journal.pone.0168348 (PMC5173368; doi:10.1371/journal.pone.0168348)
Supplement: S1 Dataset — (ZIP) [file pone.0168348.s002.zip › Taxa/Serra Norte/SN_2013/PES/Lista PESE-06.pdf]

CAVIDADE PESE-0006

Classe	Ordem	Fam/Outros	Gên/Outros	Espécie	S-U	U-S	Zon.
Arachnida	Acari	(Oribatida)		sp.2		X	E/P
Arachnida	Acari	Podocinidae	*Podocinum*	sp.1		X	P
Arachnida	Amblypygi	Phrynidae	*Heterophrynus*	*longicornis*	3	2	P
Arachnida	Araneae	Ctenidae		jovem	1	1	P
Arachnida	Araneae	Pholcidae	*Mesabolivar*	*aurantiacus*	X	X	P
Arachnida	Araneae	Pisauridae		jovem	3		E/P
Arachnida	Araneae	Theridiosomatidae	*Plato*	sp.1	X		P
Arachnida	Opiliones	Escadabiidae		sp.2	X		P
Arachnida	Opiliones	Sclerosomatidae	*Prionostema*	sp.1	X	X	P
Diplopoda	Polydesmida	Pyrgodesmidae		sp.1	X		P
Entognatha	Collembola	Paronellidae		sp.1	X	X	E/P
Entognatha	Collembola	Sminthuroidea		sp.1		X	P
Entognatha	Collembola	Tomoceridae		sp.1	X		E
Insecta	Blattodea	Blattidae		jovem	1		
Insecta	Coleoptera	Staphylinidae	Pselaphinae	sp.2	X		E/P
Insecta	Coleoptera	Staphylinidae	Scaphidiinae	sp.3	X		E
Insecta	Diptera	Psychodidae	*Pericoma*	sp.	X	X	E/P
Insecta	Diptera	Psychodidae	*Telmatoscopus*	sp.	X	X	E/P
Insecta	Diptera	Sphaeroceridae		sp.	X		P
Insecta	Diptera	Tipulidae	Tipulinae	sp.	X	X	P
Insecta	Heteroptera	Cydnidae	*Pangaeus*	sp.1		X	P
Insecta	Heteroptera	Reduviidae	Emesinae	jovem	1		P
Insecta	Heteroptera	Veliidae	*Paravelia*	sp.3	X		P
Insecta	Heteroptera	Veliidae	Veliinae	jovem	X		E
Insecta	Hymenoptera	Diapriidae		sp.2	X		E
Insecta	Hymenoptera	Formicidae	*Crematogaster*	sp.1	X		E
Insecta	Hymenoptera	Formicidae	*Gnamptogenys*	sp.2	X	X	E/P
Insecta	Hymenoptera	Formicidae	*Nylanderia*	sp.1	X		E
Insecta	Hymenoptera	Formicidae	*Nylanderia*	sp.3		X	E
Insecta	Hymenoptera	Formicidae	*Solenopsis*	sp.2		X	E
Insecta	Hymenoptera	Formicidae	*Wasmannia*	sp.1		X	E
Insecta	Isoptera	Termitidae	*Nasutitermes*	sp.	X		P
Insecta	Lepidoptera	Noctuoidea		sp.12	1		P
Insecta	Lepidoptera	Noctuoidea		sp.2	X		P
Insecta	Orthoptera	Phalangopsidae	*Phalangopsis*	sp.	34	71	P
Insecta	Psocoptera	Psocomorpha		jovem		X	E
Malacostraca	Decapoda	Trichodactylidae		sp.		4	E
Malacostraca	Isopoda	Philosciidae		sp.1	X		P
Malacostraca	Isopoda	Platyarthridae		sp.1		X	E
Symphyla		Scutigerellidae	*Hanseniella*	sp.1	X		P
Amphibia	Anura	Strabomantidae	*Pristimantis*	cf. *fenestratus*	3		E/P
Mammalia	Chiroptera	Phyllostomidae	*Carollia*	*perspicillata*	32	80	P
